# Adding Colchicine to the Antiretroviral Medication - Lopinavir/Ritonavir (Kaletra) in Hospitalized Patients with Non-Severe Covid-19 Pneumonia: A Structured Summary of a Study Protocol for a Randomized Controlled Trial

**DOI:** 10.1186/s13063-020-04455-3

**Published:** 2020-06-05

**Authors:** Nooshin Dalili, Alireza Kashefizadeh, Mohsen Nafar, Fatemeh Poorrezagholi, Ahmad Firouzan, Fariba Samadian, Shiva Samavat, Shadi Ziaie, Somayeh Fatemizadeh

**Affiliations:** 1 Department of Nephrology, Shahid Labbafinejad Medical Center, Shahid Beheshti University of Medical Sciences, Boostan 9th St., Pasdaran Av, Tehran, Iran; 2 Chronic Kidney Disease Research Center, Shahid Beheshti University of Medical Sciences, Tehran, Iran; 3 Department of Pulmonology, Shahid Labbafinejad Medical Center, Shahid Beheshti University of Medical Sciences, Tehran, Iran; 4 Clinical Pharmacy Department, Faculty of Pharmacy, Shahid Beheshti University of Medical Sciences, Tehran, Iran; 5 Department of Internal Medicine, Shahid Labbafinejad Medical Center, Shahid Beheshti University of Medical Sciences, Tehran, Iran

**Keywords:** COVID-19, Randomised controlled trial, protocol, Colchicine, Oxidative Stress, Inflammation

## Abstract

**Objectives:**

Colchicine is a well-known drug, which has been used for years to treat a wide range of rheumatic and inflammatory disorders. It helps break the cycle of inflammation through diverse mechanisms including reducing Intereukin-6, Interleukin-8, Tumour Necrosis Factor-alpha besides controlling oxidative stress pathways which all are important and pathologic components in the clinical course and outcome of patients infected with COVID-19. This study aims to assess the anti-inflammatory effects of colchicine in non-severe hospitalized COVID-19 patients.

**Trial design:**

Prospective, randomized (1:1 ratio), double blind study with parallel group design.

**Participants:**

Hospitalized patients with positive nasopharyngeal swab for COVID-19 infection (RT -PCR) and lung Computed tomography scan involvement compatible with COVID-19 pneumonia. The patients are not severely hypoxic, do not need intubation or invasive oxygenation.

Exclusion criteria: known hypersensitivity to colchicine; known hepatic failure; estimated glomerular filtration rate (eGFR)<30 ml/min/1.73m^2^ (by the CKD-EPI Creatinine Equation for Glomerular Filtration Rate (GFR) which estimates GFR based on serum creatinine. ; kidney transplant recipients, using Digoxin, QTc >450 msec.

Participants will be recruited from inpatients at Labbafinejad Meidcal Center , Tehran, Iran.

**Intervention and comparator:**

Eligible enrolled patients will be randomized into two groups. Group A will receive the antiretroviral Lopinavir/Ritonavir (Kaletra) while group B will receive Lopinavir/Ritonavir (Kaletra) + Colchicine 1.5 mg loading then 0.5 mg twice daily orally. All patients in both groups will receive the same amounts of essential minerals, vitamins as antioxidants, and antibiotics.

Patients of both groups will be treated under optimal treatment based on the CDC and WHO guidelines and national consensus proposed in Iran including the same dosages of Lopinavir/Ritonavir, antibiotics, trace elements and antioxidants while only in group-B patients Colchicine will be added on top of this protocol.

**Main outcomes:**

Primary: Time for clinical improvement and lung CT score changes 14 days after treatment.

Secondary: 14 days after treatment

– C-Reactive Protein test x Neutrophil to Lymphocyte Ratio , Interleukin-6, malondialdehyde (MDA) levels reduction

– Percentage of patients who require supplemental Oxygen

– Mean hospital stay length

**Randomisation:**

Patients will be allocated to each group (ratio 1:1) by using an online randomization tool: http://www.graphpad.com/quickcalcs/index.cfm

**Blinding (masking):**

This will be a double-blind study in which participants and those assessing the final outcomes will be blinded to group assignment.

**Numbers to be randomised (sample size):**

Regarding the pandemic crisis and our center capacity to hospitalize confirmed COVID-19 patients, a total of 80 patients was found to be logical to be randomized into two groups of 40- patients.

**Trial Status:**

Recruitment is ongoing. Recruitment began on 20/03/2020 and the date by which the recruitment is anticipated to be completed is 30/05/2020.

**Trial registration:**

ClinicalTrials.gov Identifier: NCT04360980, registered 24/04/2020.

**Full protocol:**

The full protocol is attached as an additional file, accessible from the Trials website (Additional file 1). In the interest in expediting dissemination of this material, the familiar formatting has been eliminated; this Letter serves as a summary of the key elements of the full protocol.

## Supplementary information

**Additional file 1.** Full Protocol.

## Data Availability

The corresponding author will have access to the final trial dataset and the data will be available on reasonable request via sending email to drn.dalili@sbmu.ac.ir.

