# Supplementary material for: Adding Colchicine to the Antiretroviral Medication - Lopinavir/Ritonavir (Kaletra) in Hospitalized Patients with Non-Severe Covid-19 Pneumonia: A Structured Summary of a Study Protocol for a Randomized Controlled Trial
Source: Trials. 2020 Jun 5;21:489. doi: 10.1186/s13063-020-04455-3 (PMC7273823; doi:10.1186/s13063-020-04455-3)
Supplement: Supplementary file 1 — Additional file 1. Full Protocol. [file 13063_2020_4455_MOESM1_ESM.doc]

Adding Colchicine to Lopinavir/Ritonavir (Kaletra) in Hospitalized Patients with Non-Severe Covid-19: A Randomized Clinical Trial

Nooshin Dalili,^1,2*^Alireza Kashefizadeh^3^,Mohsen Nafar^1,2^,Fatemeh pourrezagholi^1,2^,Ahmad Firoozan^1,2^,Fariba Samadian^1,2^,Shiva Samavat^1,2^,Shadi Ziaei^4^,Somayeh Fatemizadeh^5^

1- Department of Nephrology, Shahid Labbafinejad Medical Center, Shahid Beheshti University of Medical Sciences, Tehran, Iran.

2- Chronic Kidney Disease Research Center, Shahid Beheshti University of Medical Sciences, Tehran, Iran

3- Department of Pulmonology, Shahid Labbafinejad Medical Center, Shahid Beheshti University of Medical Sciences, Tehran, Iran

4- Clinical pharmacy department, faculty of pharmacy, Shahid Beheshti University of medical sciences, Tehran, Iran

5- Department of internal medicine, Shahid Labbafinejad Medical Center, Shahid Beheshti University of Medical Sciences, Tehran, Iran

**Abstract**

Objective: Colchicine is a well-known drug, which has been used for years in a wide range of rheumatologic and inflammatory disorders. It helps breaking the cycle of inflammation through diverse mechanisms including reducing IL-6, IL-8, TNF-alpha besides controlling oxidative stress pathways which all are important and pathologic components in the clinical course and outcome of patients infected with COVID-19.This study is a prospective, randomized, double-blind study to assess the anti-inflammatory effects of colchicine in non-severe hospitalized COVID-19 patients.

Methods: Prospective, randomized, double blind study. 81 patients will be randomized in two groups (A: Lopinavir/Ritonavir and B: Colchicine + Lopinavir/Ritonavir). Patients of both groups will be treated under optimal treatment based on the CDC and WHO guidelines and national consensus proposed in Iran including the same dosages of Lopinavir/Ritonavir, antibiotics, trace elements and antioxidants while only in group-B patients Colchicine will be added on top of this protocol.

Results: Trial results will be published through peer-reviewed publications.

**Introduction**

The new outbreak of COVID-19 pneumonia has faced physicians with many challenges all around the world. Meanwhile reaching to effective treatments with lowest adverse effects seems to be a united target.

Although health care givers are trying hard in a battle to overcome new COVID-19 pneumonia, it is in fact the fight between immune system and virus. There is still no globally approved consensus on how to face the virus and handle its complications meanwhile WHO guidelines seem to be the only lightening power to direct the way and each region are now using their own modified national protocols based on these sparse disseminated documents. Structured randomized clinical trials are needed urgently in joint projects between different aspects of medicine including virology, immunology and internal medicine in order to find the answers to obstacles. Therefore, we aimed to publish the structured design and end points of this study before the completion of recruiting eligible cases.

**Background**

Colchicine is a well-known drug used safely for years in different rheumatologic and inflammatory disorders, which apart from its effectiveness in gout is considered as a biophysical tool with significant power. (1) Colchicine is known to inhibit many leukocyte functions and it an inhibitor of some cytokines (e.g. IL-6, TNF-α, IL-1, platelet-derived growth factor, and transforming growth factor-β). (2)

On the other hand colchicine induced protective effects on oxidative stress by modulating vitamin E, β-carotene and Ca^2+^-release levels first evaluated in FMF cases. (3) This makes colchicine, at least from a theoretical point of view a desirable applicant for therapeutic strategies fighting against COVID-19.

As far as we know patients infected with COVID-19 are subjected to heavy inflammatory cytokine release and immune system would soon lock down combating this volume of systemic inflammation. (4)

It is hypothesized that Colchicine may reduce the cytokine storm and slowing the slope of inflamed systemic inflammation in confirmed COVID-19 cases with lung involvement before the virus find enough time to drag patients in the non-turning point of fatality.

After taking a single p.o dose, Colchicine will be absorbed in the jejunum and early Ileum (5) with maximum plasma concentrations achieved after 1-2 hours but its peak anti-inflammatory effect would only be obvious after passing 24-48h. (6) This time lag seems to be mandatory for drug to enter and be accumulated in granulocytes in which their internal drug concentration is several times higher when comparing with plasma. Subsequently it remains within monocytes for several days after the last use. (7) Regarding adverse effects, colchicine has been safely administered in patients with gout or other rheumatologic disorders.

**Research hypothesis**

It is hypothesized that Colchicine may reduce the cytokine storm and oxidative stress and slowing the slope of inflamed systemic inflammation by reducing IL-6,IL-8 and CRP X NLR in confirmed COVID-19 cases with lung involvement.

**Patient population**

Hospitalized patients with positive nasopharyngeal swab for COVID-19 infection (RT -PCR) and lung CT scan involvement compatible with COVID-19 pneumonia, which are not severely hypoxemic without the need for intubation or taking invasive oxygenation.

**Exclusion criteria**

known hypersensitivity to colchicine; known hepatic failure; eGFR<30 ml/min/1.73m^2^ by CKD-EPI equation; QTc >450 msec; kidney transplant recipients; using Digoxin, not assigning informed consent form willingly

**Study design**

Prospective, randomized, double blind study. Enrolled patients will be randomized in two groups (A: Lopinavir/Ritonavir and B: Colchicine + Lopinavir/Ritonavir). Patients of both groups will be treated under optimal treatment based on the CDC and WHO guidelines and national consensus proposed in Iran including the same dosages of Lopinavir/Ritonavir, antibiotics, trace elements and antioxidants while only in group-B patients Colchicine will be added on top of this protocol.

Flow Chart of Randomization and Treatment Assignment

Non-severe hospitalized RT-PCR positive COVID-19 cases with lung CT involvement will assess for eligibility

Will be excluded if have any of:

- eGFR<30 cc/min/1.73m^2^

- QTc>450 msec

- History of Kidney Transplant

- Known hepatic failure

- Did not sign the consent form

- Known hypersensitivity to Colchicine

- Using Digoxin

Expected 80 cases will be allocated by online Randomization tool

40 Will be Assigned to Colchicine adds on Lopinavir/Ritonavir(Kaletra) Group

40 Will be Assigned to the Lopinavir/Ritonavir(Kaletra) Group

Data Collection on Days 1,3,5,7,10,14

And Assessment Clinical improvement and CT Involvement Score and any adverse drug-related reactions

Statistical Analysis

Treatment Arm(group A): 40 RT-PCR positive COVID-19 patients without severe hypoxemia administered Colchicine plus Lopinavir/Ritonavir (Kaletra) and usual standard treatment according to national guidelines

Control Arm(group B):

40 RT-PCR positive COVID-19 patients without severe hypoxemia receiving only Lopinavir/Ritonavir (Kaletra) and usual standard treatment according to national guidelines

Usual standard treatment according to national guidelines which both treatment and control groups would receive included: vitamin C 3grams daily, 400 mg Thiamine, Selenium, Omega-3 500 mg daily, Vitamin A, Vitamin D, Azithromycin, Ceftriaxone

Endpoints

Primary: Time for clinical improvement and lung CT score changes 14 days after treatment

Secondary:

-CRP x NLR, LDH, IL-6, malondialdehyde (MDA) levels reduction

- Percentage of patients who will require supplemental Oxygen

-Mean hospital stay length

Sample size

Regarding the pandemic crisis and our center capacity to hospitalize confirmed COVID-19 patients

a total of 80 patients expected to be randomized in to two 40- patients groups.

**Ethics and dissemination**

Shahid Beheshti University of Medical Sciences Ethics Committee approved the trial with the ID :IR.SBMU.MSP.REC.165423 on 04/12/2020.

Investigators declare this trial has received ethical approval from the appropriate ethical committee as described above. We also clarify that all participants in this study would not be included unless signing an informed consent willingly and otherwise would be excluded.

**Trial registration number**

ClinicalTrials.gov Identifier: NCT04360980

**Conflict of interest**

No conflict of interest exists.

**Acknowledgements**

We want to thank all the nurses and medical staffs who dedicated their time and efforts for managing patients during COVID-19 pandemic.

**Funding**

This study received funding from Chronic Kidney Disease Research Centre (CKDRC), affiliated to Shahid Beheshti University of Medical Sciences. We declare that the funding body has no role in the design of the study and collection, analysis, and interpretation of data and in writing the manuscript.

**References**

1-Malawist, S. Colchicine, a common mechanism for its anti-inflammatory and anti-mitotic effects

Arthritis and rheumatism. Vol XI. No 2, Part 1.1968

2-Entzian, P., Schlaak, M., Seitzer, U. *et al.* Antiinflammatory and Antifibrotic Properties of Colchicine: Implications for Idiopathic Pulmonary Fibrosis . *Lung* 175 **,**41 –51 (1997).

3-Şahin, M., Cihangir Uğuz, A., Demirkan, H. *et al.* Colchicine Modulates Oxidative Stress in Serum and Leucocytes from Remission Patients with Family Mediterranean Fever Through Regulation of Ca^2+^Release and the Antioxidant System. *J Membrane Biol* 240**,**55–62 (2011).

4-Vaninov, N.In the eye of the COVID-19 cytokine storm. *Nat Rev Immunol* 20**,**277 (2020).

5- Cerquaglia C, Diaco M, Nucera G, La Regina M, Montalto M, Manna R. Pharmacological and clinical basis of treatment of Familial Mediterranean Fever (FMF) with colchicine or analogues: An update. Curr Drug Targets – Inflamm Allergy. 2005;4(1):117e124

6- Chappey ON, Niel E, Wautier JL, et al. Colchicine disposition in human leukocytes after single and multiple oral administration. Clin Pharmacol Ther.

1993;54(4):360e367

7- Molad Y. Update on colchicine and its mechanism of action. Curr Rheumatol Rep. 2002;4(3):252e256.
